# Psychotic symptoms and its association with substance use disorders among adult prisoners in correctional institution: a facility based cross-sectional study in Southwest Ethiopia

**DOI:** 10.4314/ahs.v22i1.31

**Published:** 2022-03

**Authors:** Arefayne Alenko, Habtamu Kerebih

**Affiliations:** 1 Department of Psychiatry, Faculty of Medicine, Institute of Health, Jimma University, Jimma, Ethiopia; 2 Department of Psychiatry, School of Medicine, College of Medicine and Health Sciences, University of Gondar, Gondar, Ethiopia

**Keywords:** Psychotic symptoms, substance use, prisoners, Ethiopia

## Abstract

**Introduction:**

The prevalence of psychotic symptoms among prisoners is increasing rapidly throughout the world. It imposes considerable personal and public health burden. In recent years psychotic symptoms among prisoners has been widely emphasized and the current study aimed to assess psychotic symptoms and its association with substance use disorders among adult prisoners in correctional institution in Southwest Ethiopia.

**Method:**

Facility based cross-sectional study design was conducted in Jimma Correctional Institution among 336 prisoners selected by systematic random sampling method in June 2017. Data was collected by face to face interview using structured questionnaire. Data was analyzed using SPSS version 21.0. Multivariable logistic regression was computed to identify independent associated factors.

**Results:**

The prevalence of psychotic symptoms among prisoners was found to be 43%. Poor social support (AOR: 4.12, 95%CI: 1.39–12.66), alcohol use disorder (AOR: 4.03, 95%CI: 1.58–10.27), stressful life events (AOR: 2.19, 95%CI: 1.14–4.21), and common mental disorders (AOR: 5.53, 95%CI: 2.56–11.91) were independently associated with single psychotic symptom.

**Conclusion:**

This study showed high prevalence of psychotic symptoms. Psychotic symptoms were significantly associated with poor social support, alcohol use disorder, stressful life events and common mental disorders. It is essential to have screening mechanism and management practice for psychotic symptoms.

## Introduction

Psychotic disorders such as schizophrenia and schizoaffective disorders are characterized by psychotic symptoms like delusions, hallucinations, and disorganized speech. These disorders often run a chronic or recurrent course, result in distress and functional impairment to the individual, and adversely affect a sufferer's family and community^1^. Psychotic symptoms have a lower prevalence than mood and anxiety symptoms, yet they impose a considerable personal and public health burden because of their impact on sufferers and their families^2^. One issue that has received considerable attention in recent years has been psychiatric morbidity like substance use disorders and psychotic symptoms in local jails and correctional institutions^3^,^4^,^5^. Clinical research has revealed high rates of substance use disorders, and of more concern, high rates of tobacco or nicotine among patients with psychotic symptoms6. Alcohol, cigarette (nicotine) and khat use disorders are also commonly reported among prisoners with psychotic disorders who are getting treatment in psychiatric clinic^7^,^8^,^9^. Psychotic symptoms co-morbid with substance use disorder has been associated with symptom worsening or relapse, homelessness, conflict and finally end up with imprisonment^6^,^9^. People with psychotic illness use different psychoactive substance for self-medication which results in worsening of the illness and high dose of medication than needed^2^. Incarceration in prisons is common occurrences for people with psychotic symptoms and co morbid substance use disorders, which exacerbate their marginalization and instability. Prisoners with psychotic symptoms misbehave due to the illness and allowed for disciplinary infractions at higher rates than other prisoners^10^. In developing countries like Ethiopia, people with psychosis are inappropriately locked up in prisons simply due to lack of mental health services. Adults with psychotic symptoms who commit minor offences are often sent to prison rather than treated for their illness. Psychotic symptoms therefore continue to go unnoticed, undiagnosed and untreated in prisons^11^,^12^. These cumulative effects result in increment of prison population rate in 2016, which reached 124 per 100,000 in Ethiopia^13^.

Psychotic symptoms co morbid with substance use disorder significantly impairs work, family and social functioning^13^. People with psychotic symptoms are also 4 times more likely to be unemployed or partly employed, one-third more likely not to have graduated from high school and 3 times more likely to be divorced^10^. These factors highly contribute to joblessness and involvement in criminal activities in order to get money to buy psychoactive substances. These adults finally end up with imprisonment. The evidence from different studies shows, psychotic symptoms and substance use disorder like cigarette, alcohol and khat were increased and co-morbidity is so common as to be typical of the prison population^3^,^4^,^5^,^10^. Prisoners have a high rate of suicides and self-injury due to untreated psychotic illness and situational difficulties in prisons^14^.

A 2006 survey carried out by the US Department of Justice Statistics reported that prisoners with one psychotic symptom were 11.1% in state prison and 16.8% in local jail. Those with two psychotic symptoms were 4.2% in state prison and 7.2% in local jails. Prisoners with delusions were 11.8% in state prison and 17.5% in local jails. Those with hallucination were 7.9% in state prison and 13.7% in local jails^10^. The British National Survey of Psychiatric Morbidity in 2005 reported, hallucinations (11%) and delusions (5%)^15^. The cross-sectional study conducted to assess prevalence of psychiatric morbidity among inmates in Jos maximum security prison in Nigeria in 2013; reported that the prevalence of psychosis is 1.2 %16. The cross-sectional study conducted using PSQ among street homeless people in Addis Ababa; Ethiopia reported that the prevalence of psychosis is 41.0%^17^. Apart from these, mental health is one of the most disadvantaged health programmers' in Ethiopia, both in terms of basic services and skilled manpower especially among prison population^12^.

Even though there are studies done in prevalence of psychotic disorders and its association with substance use disorders commonly schizophrenia in health institutions of Ethiopia, there is no published study as an authors' knowledge, conducted in correctional institutions. Researches on this area are based on relatively small samples and lifetime substance use. Additionally many studies have a limited ability to examine the effects of confounding factors like imprisonment and clinical factors. Therefore the current study assessed the prevalence of psychotic symptoms and its association with substance use disorders among prisoners in Jimma Correctional Institution.

## Materials and methods

A facility based cross-sectional study design was used. Data was collected from June 1- July 30, 2017. This was part of a study among prison population in Jimma town, southwest Ethiopia on research topics published elsewhere related to substance use disorder18, common mental disorders19 and psychosis. The current article focused on psychosis and its association with substance use. The sample size was determined using single population proportion formula taking p=50% at 95% CI and 5% margin of error. Since the total population in the prison was 1460 (less than 10,000) a correction formula was used and 10% non-response rate added to get a total of 336 study participants. Study participants were selected from the total of 1460 prisoners eligible for the study by a systematic random sampling technique, i.e., one participant was randomly selected from every four consecutive admissions in the registration book.

A structured questionnaire was used for the face to face interview. The questionnaire contained Socio-demographic questionnaire to assess the prisoners' background information. The psychosis screening questionnaire (PSQ) was used to determine the presence or absence of psychotic symptoms. It was assessed by endorsement of at least one psychotic symptom in Psychosis Screening Questionnaire: thought interference, paranoia, strange experience or hallucinations. The hypomania component of this tool was excluded since the aim of the study is to assess psychotic symptoms. This tool has sensitivity of 96.9%, specificity of 95.3%^20^. The Inter-rater reliability (κ) of PSQ in this study is 0.87. The Self Reporting Questionnaire 20 (SRQ-20) was used to screen the presence of common mental disorders (CMD). This tool was developed by WHO primarily to screen CMD in developing countries. The total SRQ-20 score of greater than 7 was considered as having CMD21. Inter-rater reliability (κ) of SRQ in this study is 0.79. The Oslo-3 three item scale (OSS-3) was used to assess level of social support. Based on Oslo social support scale; prisoners who scored 3–8 had poor support, 9–11 had moderate support and 12–14 had strong support^22^. In this study, Inter-rater reliability (κ) of OSS-3 is 0.820. The Life Events Checklist for DSM-5 (LEC-5) was used to screen stressful life events. Based on Life Event Checklist of DSM-5; Prisoners having at least one stressful life event^23^. Tools used to assess substance use disorder include: Alcohol Use Disorder Identification Test (AUDIT) for alcohol use disorder was used. For alcohol use disorder total AUDIT score of > 8 was used^24^. Drug Abuse Screening Test (DAST) is adapted to assess khat abuse and total DAST score of > 1 indicated khat abuse^25^, Fagerstrom Test for Nicotine Dependence (FNDT) was used to assess tobacco dependence at a FNDT score of > 3 26. The last one year use of cannabis was assessed since the prisoners have no access to use cannabis inside the correctional institution. The presence of chronic physical illness/span>, history of family and personal mental illness, history of medication use and psychotropic medication use were assessed using ‘yes or No’ answer from prisoners. The questionnaire was translated to local languages Amharic and Afan Oromo and back to English by independent person to check for consistency and understandability of the tool. The questionnaire was pretested one week prior to the actual data collection on 10% in Agaro prison center (a nearby town to Jimma) for clarity of questions. Based on this some modification were done. Six data collectors and two supervisors who have psychiatry background were selected from different institutions and were given training for three days.

### Statistical analysis

The data were entered in to EpiData (version 3.1, CDC, Atlanta, GA, USA) then exported to SPSS 21 version statistical software for analysis. Socio-demographic characteristics of respondents were analyzed by descriptive statistics (percentage, mean and standard deviations). Bivariate analysis was undertaken to identify candidate variables for the final multiple logistic regression model and variables with p-value less than 0.25 were taken as eligible for the final model. Finally multiple logistic regression analysis was conducted and significance was declared at p-value<0.05 with 95% confidence interval. Adjusted odds ratio (AOR) was used to interpret significantly associated variables. Results were presented in the form of tables.

### Ethical statement

The study was conducted after ethical clearance was obtained from the ethical review board of Institute of Health in Jimma University. Then official letter from the postgraduate coordinating office of institute of health was written to head office of prison administration. Written informed consent was obtained from each participant prisoners. Confidentiality was ensured to all participants. Prisoners with paranoia and hallucination who are in need of mental health care were linked to psychiatric clinic in Jimma University Medical Center.

## Results

### Socio-demographic characteristics

The response rate the study was 319(95%). The reason for non-response was due to refusal and interruption of interviews without fully responding to questions. Out of 319 respondents, majority 298(93.4 %) were males. The mean (± SD) age of respondents was found to be 30.40 (±11.60) year with minimum and maximum age of 18 and 86 years respectively. More than half of respondents were single 170(53.3%) in marital status and Muslim 177 (55.5%) in religion while the majority 204 (63.9%) were Oromo in ethnicity. Majority 204 (63.9%) of the respondents live in urban area prior to imprisonment and more than half 173 (54.2%) of them had attended primary education. (Table 1)

**Table 1:** Socio-demographic characteristics of prisoners in Jimma Correctional Institution; Southwest Ethiopia, 2017 (n=319)

Variables		Frequency	Percentage
**Sex**	Male	298	93.4
	Female	21	6.6
**Age**	18–27	167	52.4
	28–37	94	29.5
	38–47	35	11.0
	>=48	23	7.2
**Marital Status**	Single	170	53.3
	Married	123	38.6
	Other(divorce/widowed)	26	8.2
**Religion**			
	Muslim	177	55.5
	Orthodox	106	33.2
	Protestant	36	11.3
**Ethnicity**			
	Oromo	204	63.9
	Amhara	51	16.0
	Dawro	13	4.1
	Other	51	16.0
**Residence**			
	Urban	204	63.9
	Rural	115	36.1
**Educational** **status**	No formal education	44	13.8
	Primary education	173	54.2
	Secondary education	75	23.5
	Higher education	27	8.5
**Occupation**	Farmer Private work	83 107	26.0 33.5
	Government worker	32	10.0
	Student	37	11.6
	Labor worker	34	10.7
	Other	26	8.2
**Level of Social** **Support**	Poor support	157	49.2
	Moderate support	97	30.4
	Strong support	65	20.4

**Note:** 1month = 30 days

### Imprisonment related factors of prisoners in Jimma Correctional Institution

Among prisoners, 33(10.3%) had solitary confinement. Majority of prisoners, 232(72.7%) had no work in the prison and 266(83.4%) committed violent crimes. Regarding court's decision, 293(91.8%) was sentenced and 37(11.6%) had history of prior incarceration. (Table 2)

**Table 2:** Prison related factors of prisoners in Jimma Correctional Institution, Southwest Ethiopia 2017. (n=319)

Variables		Frequency	Percentage
**Solitary**	No	286	89.7
**confinement**	Yes	33	10.3
**Work in prison**	No	232	72.7
	Yes	87	27.3
**Type of crime**	Violent	266	83.4
	Nonviolent	53	16.6
**Court's decision**	Remand	26	8.2
	Sentenced	293	91.8
**Prior incarceration**	No	282	88.4
	Yes	37	11.6
**Stay in prison**	<12months	195	61.1
	13–24months	52	16.3
	25–36months	40	12.5
	>36months	32	10.0
**Duration of** **sentence**	<13months	78	24.5
	14–36months	84	26.3
	37–96months	80	25.1
	>97months	77	24.1

### Substance use disorders among prisoners in Jimma Correctional Institution

Out of the total 319 study respondents, 113(35.4%) had khat abuse, 51(16%) had alcohol use disorder and 31(9.7%) had nicotine dependence. About 20(6.3%) used cannabis in the last one year.

### Clinical factors among prisoners in Jimma Correctional Institution

Out of the total 319 study participants, 53(16.6%) of the respondents reported at least one chronic physical illness. About 51 (16.0%) of the respondents reported family history of mental illness, 32(10.0%) had past history of mental illness and 29(9.1%) had past admission to psychiatric clinic. About half 162(50.8%) of the respondents had CMD. Among study participants, 126(39.5%) reported one or more stressful life events. (Table 3)

**Table 3:** Clinical factors among prisoners in Jimma Correctional Institution, Southwest Ethiopia 2017(n=319)

Variables		Frequency	Percentage
Chronic Physical illness	No	266	83.4
	Yes	53	16.6
Common Mental Disorder	No	157	49.2
	Yes	162	50.8
Family Mental Illness	No	268	84.0
	Yes	51	16.0
Past Mental Illness	No	287	90.0
	Yes	32	10.0
Past admission to psychiatric clinic	No	290	90.9
	Yes	29	9.1
Currently taking psychotropic medication	Yes	25	7.8
	No	294	92.2
Stressful life event	No	193	60.5
	Yes	126	39.5

### Prevalence of psychotic symptoms among prisoners in Jimma Correctional Institution

The prevalence of psychotic symptom among prisoners in Jimma correctional institution is found to be 43(13.5%, 95%CI: 9.7–17.9%). The commonest psychotic symptoms among prisoners were paranoia 44(13.8%) and thought interference 29(9.1%). (Table-4)

**Table 4:** Prevalence of psychotic symptoms among prisoners in Jimma Correctional Institution, Southwest Ethiopia (n=319)

Psychotic symptoms	Frequency	Percent	Std. Error	95% CI	
				Lower	Upper
**Number of psychotic symptoms**					
1	43	13.5	1.9	9.7	17.9
2	18	5.6	1.3	2.8	8.2
3	7	2.2	8	0.6	4.1
4	6	1.9	0.8	0.6	3.8
**Type of psychotic symptoms present**					
Paranoia	44	13.8	2.1	10.0	17.9
Thought interference	29	9.1	1.5	6.3	12.2
Strange experience	28	8.8	1.6	6.0	12.5
Hallucinations	23	7.2	1.4	4.7	10.3

### Factors Associated with Psychotic symptoms and its associated factors among prisoners in Jimma Correctional Institution

The factors which are significantly associated with psychotic symptom in bivariate logistic regression analysis with p<0.25 were entered to multiple logistic regression analysis. These variables are candidates for multiple logistic regression after checking the assumptions (multicollinearity: VIF<10, and large sample). The Hosmer Lemeshow goodness of fit statistic (p=0.712) and loglikelihood statistics were used to assess whether the necessary assumptions for the application of multiple logistic regression are fulfilled.

In multiple logistic regression analysis: poor social support, alcohol use disorder, having stressful life events and CMD were significantly associated with psychotic symptoms (p<0.05). The odds of having psychotic symptoms was 4 times (AOR=4.12 CI: 1.39–12.66) higher among prisoners with poor social support than the odds of moderate and strong social support. The odds of having psychotic symptoms was 4 times (AOR=4.03(1.58–10.27) higher among prisoners alcohol use disorder. The odds of having psychotic symptoms was 2.20 times (AOR=2.19(1.14–4.21) higher among prisoners who had one or more stressful life events. The odds of having psychotic symptoms was 5.53 times (AOR= 5.53(2.56–11.91) higher among prisoners with CMD. (Table 5)

**Table 5:** Multivariate Logistic regression analysis of factors associated with psychotic symptom among prisoners in Jimma Correctional Institution Southwest Ethiopia, 2017 (n=319)

Variables		COR (95% C.I.)	AOR (95% C.I.)	P value
Level of Social Support	Poor	4.20(1.69 – 10.40)	4.12(1.39–12.66)	.011*
	Moderate	2.71 (1.03–7.16)	2.627(0.82–8.38)	.043
	Strong	1.00	1.00	

Solitary Confinement	Yes	2.06(0.96–4.42)	1.33(.46–3.83)	.593
	No	1.00	1.00	
Work in Prison	No	0.56(0.32–0.98)	1.92(.91–4.02)	.083
	Yes	1.00	1.00	

Alcohol use disorder	Yes	3.50(2.86–6.57)	4.03(1.58–10.27)	.003*
	No	1.00	1.00	

Nicotine Dependence	Yes	3.13(1.46–6.70)	1.25(.37–4.17)	.709
	No	1.00	1.00	
Khat Abuse	Yes	1.92(1.13–3.26)	.94(.41–2.12)	.886
	No	1.00	1.00	

Cannabis use in the last one year	Yes	5.73(2.24–14.63)	2.02(.55–7.45)	.288
	No	1.00	1.00	

Stressful Life events	Yes	3.14(1.83–5.37)	2.19(1.14–4.21)	.019*
	No	1.00	1.00	

Family Mental Illness	Yes	2.55(1.35–4.83)	1.00(.42–2.40)	.991
	No	1.00	1.00	

Chronic Physical illness	Yes	3.23(1.73–6.02)	1.23(.50–2.97)	.645
	No	1.00	1.00	

Past Mental Illness	Yes	5.30(2.48–11.30)	.402(.03–4.40)	.456
	No	1.00	1.00	

Past Admission to psychiatric clinic	Yes	6.79(2.61–12.80)	1.19(.06–21.40)	.903
	No	1.00	1.00	

Taking psychotropic medication	Yes	7.23(3.04–17.19)	13.83(.76–250.70)	.076
	No	1.00	1.00	

CMD	Yes	4.47(2.45–8.12)	5.53(2.56–11.91)	.000**
	No	1.00	1.00	

Note: 1.00=constant **=p<0.001 *=p<0.05 CMD=Common Mental Disorders

Model: Chi-square=94, df = 21, and Sig. <0.001.

## Discussion

Untreated psychotic symptoms end up with a long-standing morbidity, stigma and mortality due to suicide and infectious diseases. It also causes psychological trauma for families, friends and relatives of individual with psychotic symptoms. The cumulative effects result in loss of economic productivity for the nation. The underlying reason for assessing psychotic symptoms in correctional institutions is to ensure that treatment plans and evaluations focus on prisoners' risk factors.

The past one year prevalence of single psychotic symptom among prisoners in Jimma correctional institution was 13.5%. These finding is nearly two times higher than Kenyan community based study^27^ and four times higher than Tanzanian community based study^28^. The higher prevalence of psychotic symptoms among prisoners compared to the general population is reported in many studies around the world^10^,^14^. The prevalence of psychotic symptoms in the past one year is found to be paranoia (13.8%), thought interference (9.1%), strange experience (8.8%) and hallucinations (7.2%)The higher prevalence of paranoia and hallucinations in this study when compared to studies conducted in US 10 and Britain^15^ is due to socio-economic difference between the study populations. The other possible reason could be due to the assessment tools used, PSQ which is primarily psychotic screening tool was used in the current study whereas, but the previous studies used clinical diagnostic tools.

Prisoners having poor social support were four times more likely to develop psychotic symptoms. This finding agrees with the study conducted in Turkey in which people with poor social support were four and half times more likely to develop psychotic symptoms^29^. Similarly, self-reported psychotic symptoms using PSQ in Britain were significantly associated with small primary support group (few close friends or relatives)^30^. It is plausible that poor social support in addition to social isolation due to imprisonment might contribute to the development of negative schemas in these prisoners as a psychosocial risk factor. Prevalence of psychotic symptoms using PSQ was elevated most significantly among immigrants living in Italy with poor social support and having poor social integrity^31^. This might be due to features of positive symptoms, which cause individuals to withdraw from social networks or create difficulty in maintaining relationship as a result of realty impairment.

This study found that prisoners with alcohol use disorder were four times more likely to have psychotic symptoms. Similarly, it has been reported that alcohol use is five times more risk for psychotic symptoms compared to people who had no use of alcohol^28^,^29^. The possible mechanism is that alcohol is related to dopamine mediated brain reward pathways in the mesocorticolimbic tracts^32^ and induced psychotic symptoms by increasing dopamine in the mesolimbic areas of the brain of people whose brains had been sensitized due to the genetic and neurodevelopmental factors^7^.

Prisoners having one or more stressful life events were 2.2 times more likely develop psychotic symptoms than prisoners not having stressful life events. Studies shows psychotic symptoms were associated with stress related to relationship problems, manmade and natural disaster, employment, financial crises and victimization experiences^28^,^5^. The possible mechanism is that stressful events are regarded as an important component influencing the biological functioning of the brain. This is due to stress induced abnormally activated HPA axis causes neurotoxicity in the central nervous system. This effect is related to the increased level of the glucocorticoid hormones^33^.

Prisoners having CMD in SRQ-20 were about five and half times more likely to develop psychotic symptoms than who have no CMD. This finding agrees with the study conducted in Kenya^27^ and Tanzania^28^. Similarly in British study, the self-reported psychotic symptoms were significantly associated with CMD^30^. In a study of adult primary care patients, psychotic symptoms were associated with anxiety and depression, and these were all clinical consequences of psychotic symptoms^34^. On the other hand, CMD have been reported in excess in those adults who later develop psychotic symptoms^35^. Due to this, it had been identified as part of the initial prodromal in psychotic illness.

## Strength and Limitation of the study

The tools we used were standardized and internationally recognized screening tools with high reliability to screen psychotic symptoms and substance use disorders. Recall bias and social desirability bias due to the nature of the questions and data collection methods should be taken in to considerations.

## Conclusion

High prevalence of Psychotic symptoms was found in this study. Among substances, Alcohol use disorders was significantly associated with psychotic symptoms and other psycho-social factors include poor social support, common mental disorders and one or more stressful life events. Therefore, psychotic symptoms are major public health problem in Jimma Correctional Institution. To improve the mental health status of the prisoners, there is a need to psychotic symptom screening mechanism and management practice in correctional institutions which involves all concerned bodies. Further research is recommended to assess prevalence of psychotic disorders by using standardized clinical diagnostic tools at correctional institution in the country.

## Data Availability

The data could be available upon reasonable request.
